# Conversion surgery for advanced jejunal adenocarcinoma with multiple peritoneal metastases: a case report

**DOI:** 10.1186/s40792-023-01716-6

**Published:** 2023-08-17

**Authors:** Miku Obayashi, Shimpei Otsuka, Ryo Ashida, Katsuhisa Ohgi, Mihoko Yamada, Takeshi Kawakami, Katsuhiko Uesaka, Teiichi Sugiura

**Affiliations:** 1 Division of Hepato-Biliary-Pancreatic Surgery, Shizuoka Cancer Center, 1007 Shimo-Nagakubo, Nagaizumi-Cho, Sunto-Gun, Shizuoka, 411-8777 Japan; 2 Division of Gastrointestinal Oncology, Shizuoka Cancer Center, 1007 Shimo-Nagakubo, Nagaizumi-Cho, Sunto-Gun, Shizuoka, 411-8777 Japan

**Keywords:** Jejunal adenocarcinoma, Small bowel cancer, Peritoneal metastases, Conversion surgery, Complete response, FOLFOX, Chemotherapy, Multimodal therapy

## Abstract

**Background:**

Small bowel cancer (SBC) is a rare malignancy that is often diagnosed at an advanced stage. Palliative chemotherapy is the standard treatment for patients with metastatic SBC. The relevant literature on conversion surgery in patients who have responded favorably to chemotherapy is limited.

**Case presentation:**

A 64-year-old man was diagnosed with jejunal carcinoma with multiple peritoneal metastases. After implanting an expandable metallic stent at the primary site, the patient underwent 6 months of FOLFOX therapy, resulting in a clinical complete response. Chemotherapy was continued, and four years after the initiation of therapy, the patient showed no evidence of disease progression. Nevertheless, anemia of continuous minor hemorrhages from the stent site and general malaise of chemotherapy got progressively worse during treatment. After confirming negative ascites cytology and the absence of peritoneal metastasis via staging laparoscopy, the patient underwent partial jejunectomy. Pathologically, no residual tumor was detected in the resected specimen. The postoperative course was uneventful, and the patient remained free of recurrence for 30 months after surgery without chemotherapy.

**Conclusion:**

Although infrequent, conversion surgery may be a valid therapeutic option for selected cases of SBC with peritoneal metastasis.

## Background

Small bowel cancer (SBC) is a rare neoplasm, comprising approximately 1% of all gastrointestinal malignancies [1, 2]. The incidence of SBC has been slowly increasing in recent years [3]. Most SBC occurs in the duodenum, and only about 30% originate in the jejunum [4]. Unfortunately, SBC tends to be diagnosed at an advanced stage in comparison to colorectal carcinoma (CRC) due to difficulties in screening for this condition, and approximately one-third of SBC patients were initially diagnosed with distant metastases [3]. Furthermore, peritoneal metastasis is reported to be detected in 20–50% of patients and is particularly common in jejunal and ileal carcinoma [5].

We report the case of a patient with jejunal adenocarcinoma with peritoneal metastases who underwent conversion surgery (CS) after chemotherapy and achieved 30 months of relapse-free survival after surgery.

## Case presentation

**Fig. 1:**
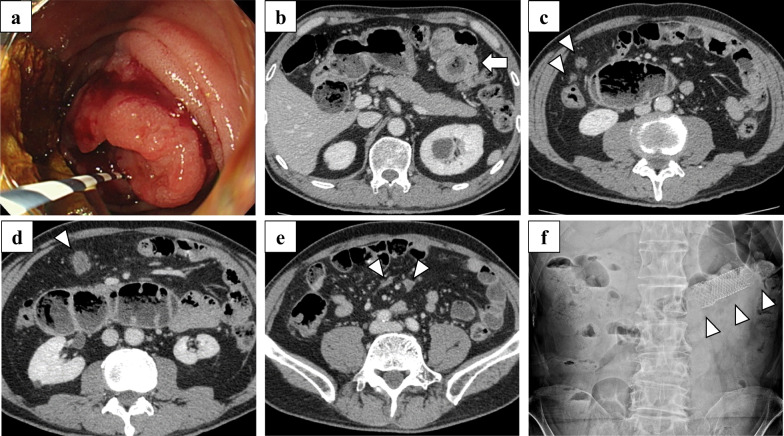
Images before chemotherapy. **a** Jejunal examination with an endoscope detected a circumferential hemorrhagic tumor in the upper jejunum. **b** Computed tomography revealed a tumor of the jejunum (arrow). **c**–**e** Multiple peritoneal metastases were observed spanning from the epigastric region down to the lower abdominal area (arrowheads). The larger peritoneal metastases were primarily located in the greater omentum (**c**, **d**). **f** An expandable metallic stent (Boston WallFlex^®^ Duodenal Stent 22 × 60 mm, arrowheads) was implanted and expanded

A 64-year-old man presented with abdominal pain and melena and underwent a jejunal examination with an endoscope which revealed a 2-cm length circumferential hemorrhagic tumor in the upper jejunum (Fig. 1a). A biopsy confirmed the diagnosis of well-differentiated and papillary adenocarcinoma. Contrast-enhanced computed tomography revealed a tumor of the jejunum and multiple intraperitoneal nodules (Fig. 1b–e). The patient was diagnosed with jejunal adenocarcinoma with peritoneal metastasis, cT3N0M1, cStage IV (8th edition of the Union for International Cancer Control TNM classification of malignant tumors). An expandable metallic stent (Boston WallFlex^®^ Duodenal Stent 22 × 60 mm) was implanted and he was subsequently referred for chemotherapy (Fig. 1f).

**Fig. 2:**
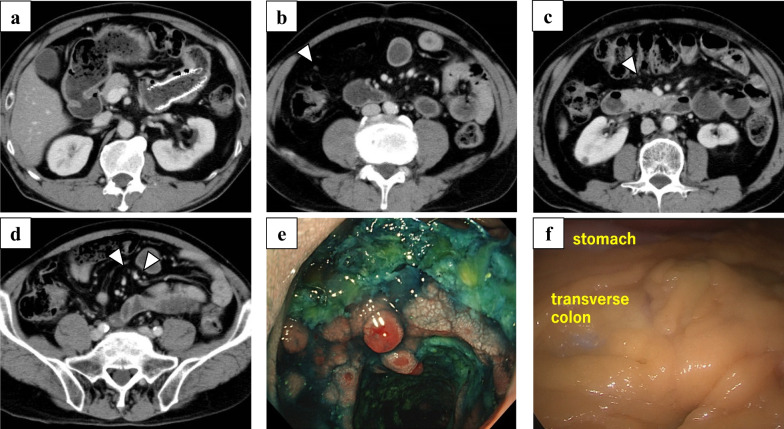
Six months after initiation of chemotherapy. **a** Computed tomography revealed the stent placed in the primary lesion, but shows no noticeable thickening of the surrounding walls. **b**–**d** Computed tomography showed extreme shrinkage of the patient’s peritoneal metastases (arrowhead). **e** Endoscopy found the growth of granulation in the stent lumen. Biopsy revealed no malignant findings. **f** The staging laparoscopy did not identify the metastatic nodules that were previously observed in the omentum

After 6 months, 11 course of palliative chemotherapy of modified FOLFOX 6 (5-fluorouracil, calcium levofolinate, and oxaliplatin), the primary lesion and peritoneal metastases were significantly reduced in CT (Fig. 2a–d), and a biopsy of the primary lesion was negative (Fig. 2e). Although the patient had achieved a clinically complete response (CR) according to RECIST classification [6], chemotherapy was continued with 5-fluorouracil and calcium levofolinate without oxaliplatin at the oncologist’s direction. Over 4 years, 92 course of chemotherapy, no disease progression was observed, although the patient experienced persistent small hemorrhages from the stent site leading to anemia. In addition, general malaise got worse and became problematic. CS was therefore planned to control these adverse events after confirming the absence of peritoneal metastases and tumor cells in ascites by staging laparoscopy (Fig. 2f).

**Fig. 3:**
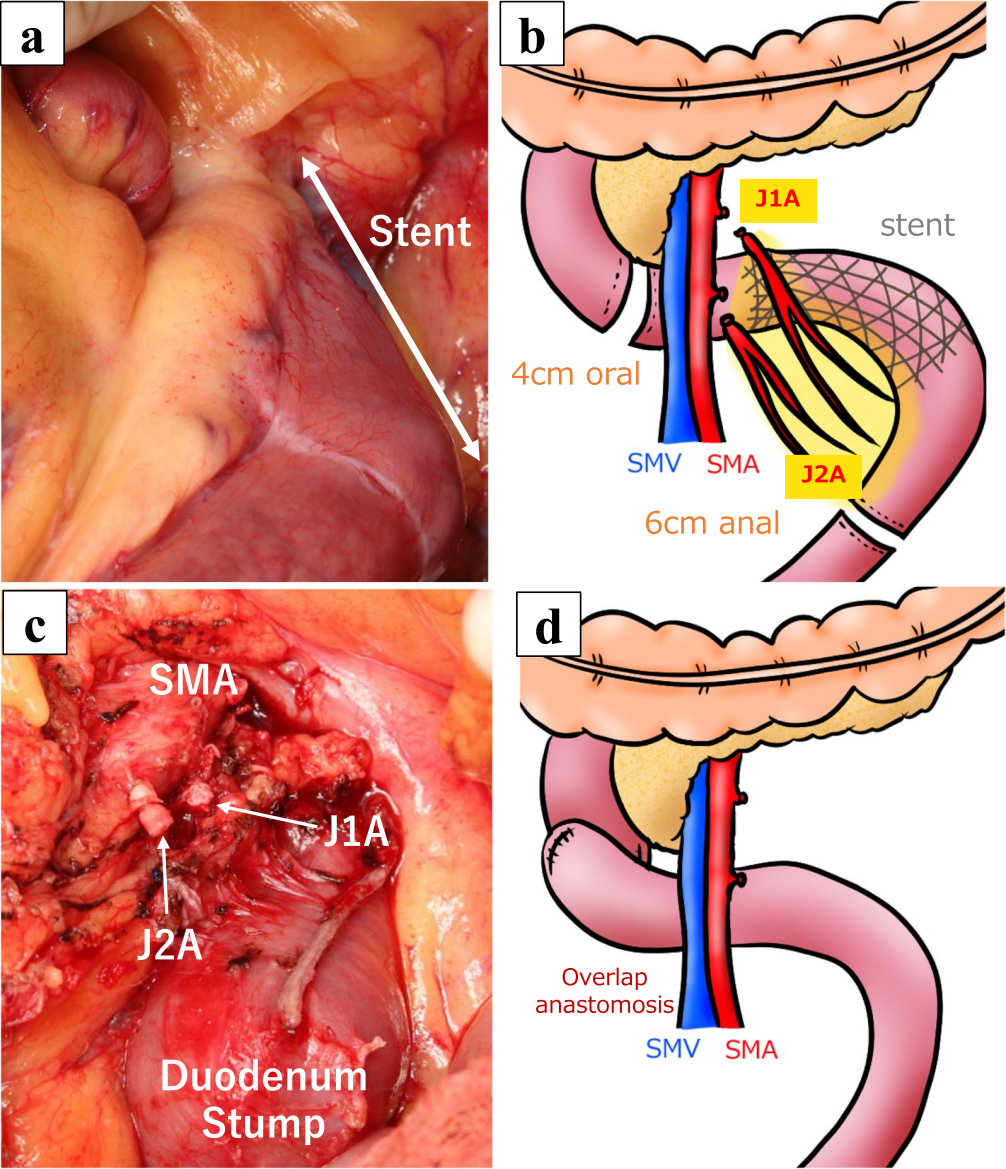
Surgical procedure. **a** Before resection. The stent was palpable, and any indication of tumor exposure to the serosa or grossly visible peritoneal metastasis. **b** Schema of resection line. The resection margin was set 4 cm on the oral side and 6 cm on the anal side of the stent. Lymph nodes in the region of J1A and J2A were dissected. **c** After resection. The J1A and J2A were transected, and the plexus of the SMA was preserved. The duodenum was dissected at the level of the 3rd portion. **d** Schema of reconstruction. Overlap anastomosis was performed. *J1A* 1st jejunal artery, *J2A* 2nd jejunal artery, *SMA* superior mesenteric artery, *SMV* superior mesenteric vein

**Fig. 4:**
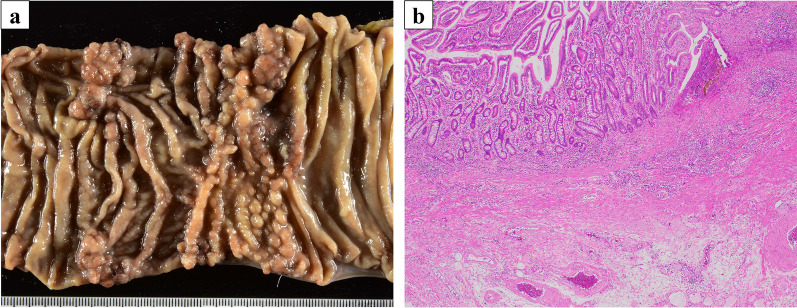
Findings of resection specimen. **a** Gross findings. No discernible tumor was identified in the stent lumen. **b** Pathological findings. A whole split specimen was prepared. Hyperplastic changes, fibrosis, granulomatous tumor changes, and small abscesses were noted, but no obvious residual cancer was observed

Partial jejunectomy was performed. An examination of the entire gastrointestinal tract and grater omentum revealed that the peritoneal metastases had vanished. After the duodenum dissected from the ligament of Treitz and the transverse mesocolon, the resection margin was set 4 cm on the oral side (3rd portion of the duodenum) and 6 cm on the anal side from the stent. Lymph nodes in the region of the first to second jejunal artery were dissected, and the plexus of the superior mesenteric artery was preserved. The jejunum was elevated through the dorsal side of superior mesenteric artery and overlap anastomosis was performed (Fig. 3a–d). The operating time was 193 min and blood loss were 301 ml. Grossly, no discernible tumor was identified in the stent lumen (Fig. 4a). Pathologically, no residual malignancy was detected, and a pathological CR was determined (Fig. 4b). The patient had no problems during his postoperative course and was discharged on the 9th postoperative day. No adjuvant chemotherapy was performed, and he was alive at 30 months after the operation without recurrence.

## Discussion

We report a case of CS after chemotherapy for jejunal carcinoma with peritoneal metastases. Chemotherapy was continued for a prolonged period after clinical CR was achieved because the clinical significance of primary tumor resection in jejunal carcinoma with multiple peritoneal metastases was uncertain. Nonetheless, the patient eventually developed anemia and general malaise, leading to resection 4 years after the initiation of chemotherapy. In a similar case, there is only one report of a patient with SBC and distant metastases who underwent CS after a successful chemotherapy [7]. The patient had liver metastases and multiple peritoneal metastases and achieved clinical CR after 12 cycles of FOLFOX + bevacizumab therapy, and the primary tumor was resected. It is noteworthy that in our reported case, clinical CR was achieved only 6 months after treatment initiation, and early resection may have been possible. However, the appropriateness and timing of CS for SBC patients who respond well to chemotherapy cannot be determined from these case reports alone. Considering the low physical burden of SBC resection, CS is a reasonable option when continuation of chemotherapy is difficult, as in our case.

The appropriate timing of CS remains a controversial topic in several types of cancer. In gastric cancer, a classification for surgical indication has been proposed to identify suitable candidates for CS [8]. The classification recommends approximately 6 months of chemotherapy, which is close to the median progression-free survival, and suggests CS for patients who achieve CR or partial response. However, the classification does not recommend CS for patients with peritoneal metastases due to the high risk of peritoneal recurrence after surgery. Nevertheless, recent reports indicate that CS may improve overall survival in patients with limited peritoneal metastases due to advances in chemotherapy [9]. In colorectal cancer (CRC), surgical resection is recommended for patients with peritoneal metastases who have no extraperitoneal metastases and are suitable for complete resection [10]. CS is not initially considered in these cases. The efficacy of CS after chemotherapy for patients with extensive peritoneal metastases that are difficult to completely resect remains uncertain due to the lack of a consensus. In pancreatic cancer, peritoneal metastasis resection is typically not performed. A retrospective study reported the use of CS following intraperitoneal paclitaxel and systemic chemotherapy for patients with peritoneal metastases [11]. Of the 79 treated patients, 16 underwent CS after re-evaluation of the indications more than 8 months after treatment initiation. Although many patients experienced recurrences after surgery, three survived more than 2 years without recurrence. To perform CS for small intestinal cancer, it is desirable to have a minimum chemotherapy duration of at least 6 months, based on experience with other carcinomas. However, further case series are needed to determine the significance of CS and identify the most appropriate timing for CS.

SBC shares molecular features with both CRC and gastric cancer, and chemotherapy has been applied similarly for both conditions. The National Comprehensive Cancer Network guidelines (Version 1.2020) provide separate treatment for metastatic duodenal and jejuno-ileal cancer. FOLFOX, CAPEOX (capecitabine and oxaliplatin), or FOLFOXIRI (5-fluorouracil, calcium levofolinate, oxaliplatin, and irinotecan) plus bevacizumab is recommended for unresectable cases of metastatic jejuno-ileal carcinoma. The guidelines also state that the objective of chemotherapy for locally advanced unresectable cases is CS, while the objective of chemotherapy for unresectable cases with distant metastasis is only palliation [12]. While the revised 2020 European Society of Gastrointestinal Endoscopy Guideline for self-expandable metal stents states that the use of bevacizumab is acceptable while a stent is in place [13], it has long been suggested that the use of bevacizumab in cases with a stent poses a risk of perforation. Considering that in this case, a CR was achieved even without the use of bevacizumab, we believe that careful judgement is required when using bevacizumab in cases with a stent. Recently, microsatellite instability (MSI) and tumor mutation burden (TMB) have been observed more frequently in SBC than CRC [14]. Either pembrolizumab or nivolumab with or without ipilimumab as the subsequent therapy are recommended for unresectable SBC with MSI-high with distant metastases [12]. In our case, the MSI status and TMB were not investigated as it was not yet a standard practice at the time of the initiation of treatment. There is an increasing trend of tailored therapies based on genetic profiles, which is expected to increase the success rate of achieving CS in case of SBC by using different chemotherapies for individuals.

## Conclusions

Although infrequent, in cases of SBC with peritoneal metastasis, CS following chemotherapy can be considered the therapeutic option for selective candidates.

## Data Availability

All data generated or analyzed during this study are included in this published article.

## Abbreviations

SBC: Small bowel cancer
CS: Conversion surgery
CR: Complete response
CRC: Colorectal carcinoma
MSI: Microsatellite instability
TMB: Tumor mutation burden
